# Correction: Blal et al. The Effect of Cannabis Plant Extracts on Head and Neck Squamous Cell Carcinoma and the Quest for Cannabis-Based Personalized Therapy. *Cancers* 2023, *15*, 497

**DOI:** 10.3390/cancers15092481

**Published:** 2023-04-26

**Authors:** Kifah Blal, Elazar Besser, Shiri Procaccia, Ouri Schwob, Yaniv Lerenthal, Jawad Abu Tair, David Meiri, Ofra Benny

**Affiliations:** 1 Department of Oral and Maxillofacial Surgery, Hadassah Medical Center, Faculty of Dental Medicine, The Hebrew University of Jerusalem, Jerusalem 9112102, Israel; 2 Department of Pharmaceutical Science, School of Pharmacy, Faculty of Medicine, The Hebrew University of Jerusalem, Jerusalem 9112002, Israel; 3 Laboratory of Cancer Biology and Cannabinoid Research, Department of Biology, Technion-Israel Institute of Technology, Haifa 3200003, Israel; 4 Cannasoul Analytics Ltd., Caesarea 3079822, Israel

In the original publication [1], there was a mistake in the Figure 8C published. The author uploaded the “dead cell” instead of the “vital cells percentage”. The corrected Figure 8C appears below.


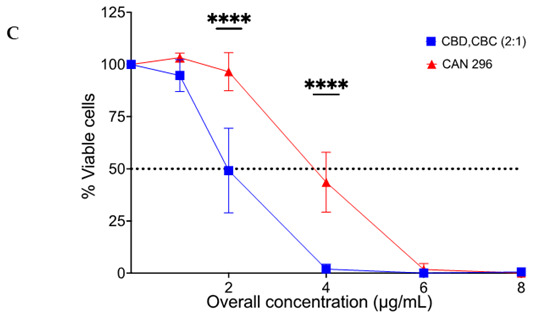


The authors state that the scientific conclusions are unaffected. This correction was approved by the Academic Editor. The original publication has also been updated.
