# Supplementary material for: The Effect of Cannabis Plant Extracts on Head and Neck Squamous Cell Carcinoma and the Quest for Cannabis-Based Personalized Therapy
Source: Cancers (Basel). 2023 Jan 13;15(2):497. doi: 10.3390/cancers15020497 (PMC9856564; doi:10.3390/cancers15020497)
Supplement: Supplementary file 1 [file cancers-15-00497-s001.zip › cancers-2139787-supplementary.pdf]

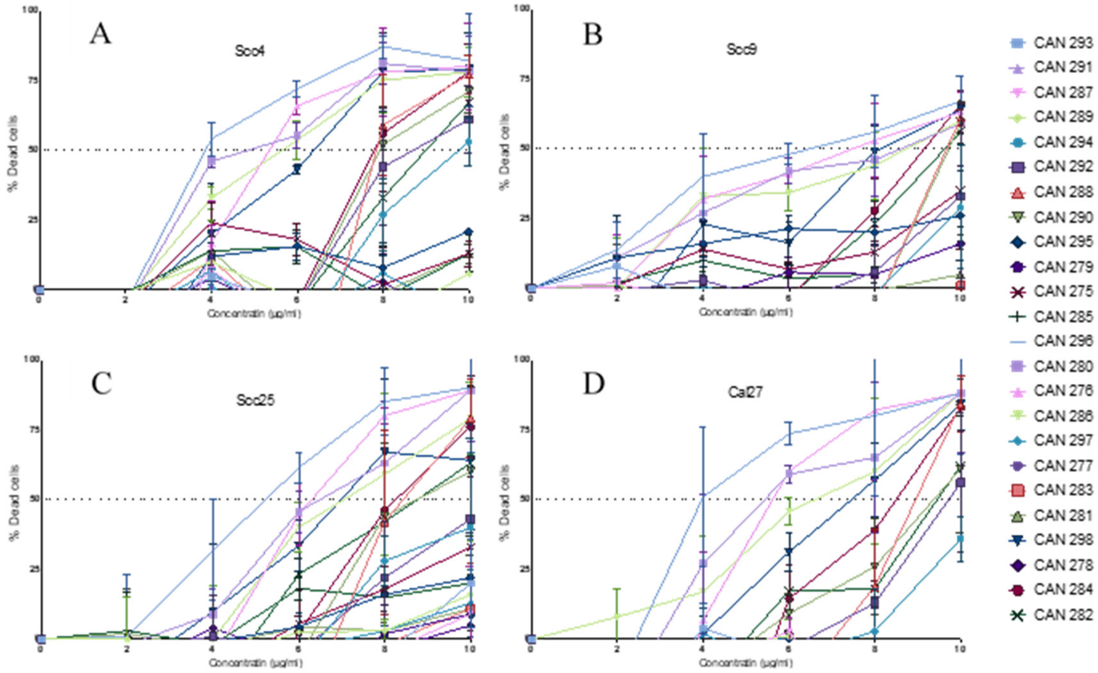


**Figure S1.** Differential effect of different Cannabis extracts on the viability of HNSCC cells. Oral SCC cell lines (A) Scc4, (B) Scc9, (C) Scc25, and (D) Cal27 were treated with 24 cannabis extracts at increasing concentrations of 2-10 µg/ml for 24 hours. Cell viability was evaluated by MTT and data are reported as mean ± SE (N=3) of % dead cells compared to DMSO control, $\% \, Dead \; cells = \frac{100x(Control - Sample)}{Control}$.

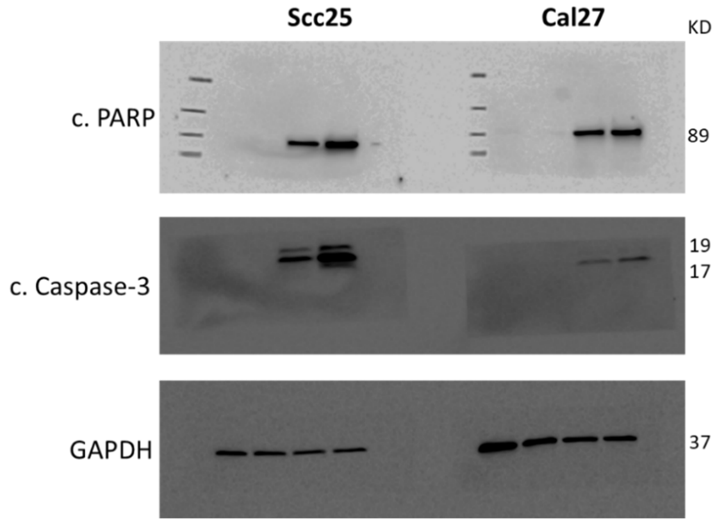


**Figure S2. Apoptotic effect of Cannabis extracts on HNSCC uncropped blots.** Scc23 and Cal27 cells were treated with Extract CAN296 (2, 4, 8 µg/ml) for 12 hours, then lysed and resolved on 15% SDS-PAGE, followed by western blotting with c.PARP, anti-Cleaved Caspase 3, and GAPDH as the loading control.
